# A case report of pseudoprogression followed by complete remission after proton-beam irradiation for a low-grade glioma in a teenager: the value of dynamic contrast-enhanced MRI

**DOI:** 10.1186/1748-717X-5-9

**Published:** 2010-02-04

**Authors:** Candice Meyzer, Frédéric Dhermain, Denis Ducreux, Jean-Louis Habrand, Pascale Varlet, Christian Sainte-Rose, Christelle Dufour, Jacques Grill

**Affiliations:** 1 Department of Pediatric and Adolescent Oncology, Gustave Roussy Institute, 39 Rue Camille Desmoulins, 94805 Villejuif, France; 2 Department of Radiation Therapy, Gustave Roussy Institute, Villejuif, France; 3 LIMEC, INSERM UMR 788, University Hospital, Kremlin-Bicêtre, France; 4 Centre de Protonthérapie, Orsay, France; 5 Department of Neuropathology, Sainte-Anne Hospital, Paris, France; 6 Depatment of Neurosurgery, Necker Sick Children's Hospital, Paris, France

## Abstract

A fourteen years-old boy was treated post-operatively with proton therapy for a recurrent low-grade oligodendroglioma located in the tectal region. Six months after the end of irradiation (RT), a new enhancing lesion appeared within the radiation fields. To differentiate disease progression from radiation-induced changes, dynamic susceptibility contrast-enhanced (DSCE) MRI was used with a T2* sequence to study perfusion and permeability characteristics simultaneously. Typically, the lesion showed hypoperfusion and hyperpermeability compared to the controlateral normal brain. Without additional treatment but a short course of steroids, the image disappeared over a six months period allowing us to conclude for a pseudo-progression. The patient is alive in complete remission more than 2 years post-RT.

## Background

The occurrence of new contrast enhancing lesions on routine MRI follow-up after radiation therapy (RT) for brain gliomas was observed more than ten years ago but remains problematic because standard MR imaging techniques do not allow a clear distinction between recurrent tumour and radiation-induced lesions [1,2]. Recently, "pseudo-progression" was defined as conventional MR images compatible with progression, occurring shortly after concomitant radio-chemotherapy (CRC), as a transient phenomenon with spontaneous improvement or stabilization after several months [3-5]. This was mainly described in adult and paediatric populations with high-grade gliomas whose new standard of care is CRC followed by adjuvant chemotherapy [6,7].

We report here a clinical case showing a new contrast-enhancing lesion discovered on a systematic MRI proposed to a fourteen years-old boy treated six months before for a recurrent low-grade glioma by surgery and proton-therapy. Dynamic Susceptibility Contrast (DSC) MR imaging and planned follow-up lead to the diagnosis of pseudo-progression. This phenomenon has rarely been described in children with supra-tentorial low-grade gliomas after proton beam RT, particularly with this type of advanced MR technique.

## Case Presentation

### Initial presentation and conventional MRI description

**Figure 1:**
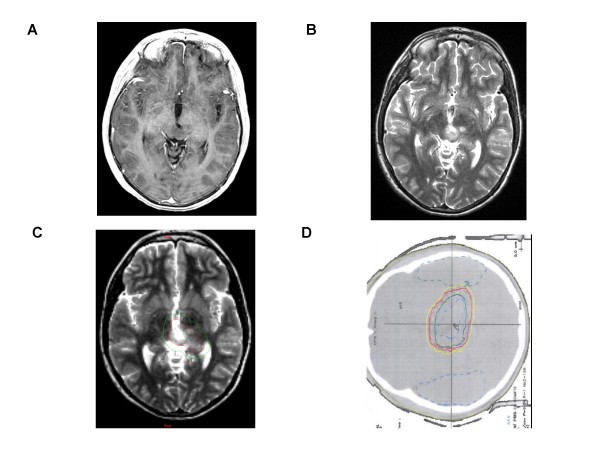
**Conventional MR imaging, Target volume and Dosimetry of a Low-grade Glioma**. A left thalamic low-grade oligodendroglioma in a 10 year-old boy after partial resection, without contrast enhancement. [A] T1-weighted transaxial MR image after administration of intravenous Gd-DTPA [B] T2 -weighted sequence which shows ill-defined nodular images. [C] Representation of Gross tumor volume (in red) and Clinical target volume (in green). [D] Radiation field encompassing medial part of the left thalamus.

**Figure 2:**
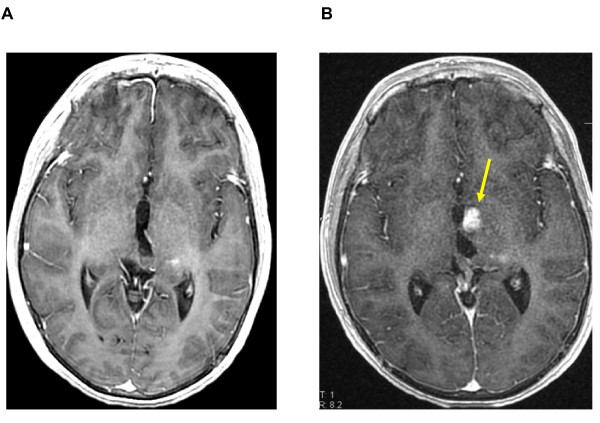
**Conventional MR imaging before and six months after exclusive Proton therapy**. [A] T1-weighted transaxial MR image after administration of intravenous (iv) Gd-DTPA, one week before radiation. [B] The same sequence, six months after the end of irradiation (54 Gy), showing a strong contrast enhancement with a subtle mass effect (yellow arrow).

A healthy ten year-old boy with tinnitus and bilateral papillary oedema underwent a brain MRI showing a small tumour of the tectal plate. The lesion presented a signal of low intensity on T1-weighted and a high signal on T2-weighted sequences. No contrast enhancement was visible. The process was responsible for triventricular hydrocephalus by aqueduct compression. The first treatment was a ventriculocysternostomy followed, fifteen months later, by a partial resection when the lesion increased in size. Histopathological diagnosis was a grade II oligodendroglioma (WHO classification), with a proliferation index (MIB 1) of 5%. Three years after the first surgery, a systematic follow-up MRI revealed a recurrence localized on the quadrigeminal plate, close to the left thalami and the third ventricle, again without gadolinium enhancement. A second surgery was attempted, showing the same histology, but a tumour residue was left on the left thalamus (Figure 1). Treatment was completed six weeks later by RT using proton beams, delivering 54 Gy in 30 fractions of 1.8 Gy. Radiation fields encompassed the medial parts of the thalami (Figure 1C, D). Radiological and clinical data were then evaluated every 3 months thereafter. Six months after RT, systematic MRI found obvious development of a new lesion with an important contrast enhancement and mass effect on the third ventricle (Figure 2), however the patient remained asymptomatic.

### Conventional and Dynamic MRI with Permeability visualization and Perfusion estimate

**Figure 3:**
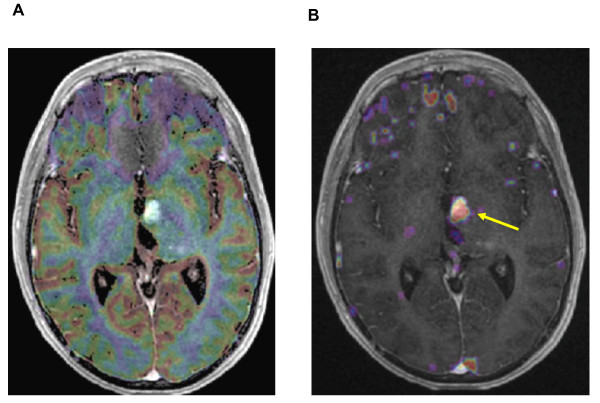
**Six months after irradiation: superimposed MR images with T1-weighted sequence after iv Gadolinium injection and co-registered Perfusion (left) and Permability maps (right)**. [A] Gradient-echo axial image with color overlay map showing no focus of hyperperfusion in regard of the strong contrast enhancement [B] The superimposed Permeability map with a strong microvascular leakage (yellow arrow) strictly corresponding with the enhanced area in the T1-weighted sequence.

**Figure 4:**
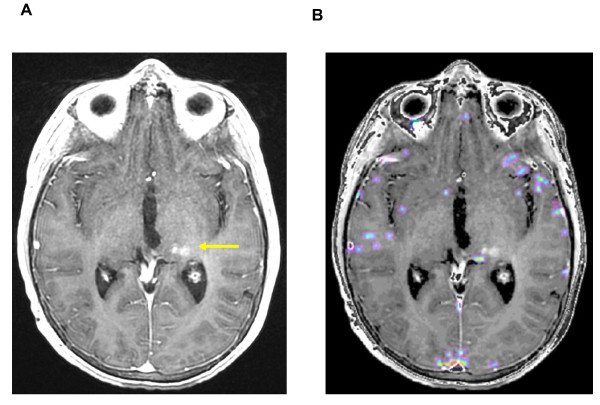
**Nine months after irradiation: complete disappearance of the contrast enhancement (left) and no detectable Permeability (right)**. [A] T1-weighted transaxial MR image after administration of iv Gd-DTPA and [B] the superimposed Permeability map, with no contrast enhancement or detectable microvascular leakage. To be noted, a subtle hypersignal, pre-existing to the irradiation, as a post-operative modification (yellow arrow).

In order to more accurately differentiate tumour recurrence from radiation-induced changes, a DSC-MRI was performed as described previously [8,9]. The T2* sequence was used in order to assess simultaneously perfusion and permeability characteristics, as recently proposed for predicting risk of recurrence in adult low-grade gliomas [10]. In this case, the typical combination of a focused area of high permeability strictly superimposed on the contrast enhancing lesion combined with the absence of high perfusion estimate in the same region (Figure 3) was compatible with post-radiation modifications, as recently suggested by Hu et al. They found a direct correlation between image-guided tissue histopathology and localized DSC perfusion MR imaging measurements in a series of adult high-grade gliomas [11]. Consequently, we favoured the hypothesis of a delayed radiation injury and began a treatment with oral steroids (prednisone) at one mg/kg/d for one month before clinical and radiological reappraisal. After one month, decrease in size of contrast enhancement became significant while the patient remained asymptomatic. Steroids were therefore discontinued. Gradual improvement continued on conventional MRI and disappearance of both contrast enhancement and high permeability area strongly suggested a pseudo-progression rather than recurrent disease. Follow-up MRI at nine months post-RT showed a complete resolution of the gadolinium uptake and no detectable microvascular leakage (Figure 4). At last follow-up, patient is still in complete remission, more than two years post-RT.

### Post-treatment modifications, evolution with time and micro-vascular hypothesis

Subacute radiation-induced modifications have been described previously in childhood gliomas, with or without concomitant or sequential use of chemotherapy [1,12-14]. In adults, these changes are more and more reported after combined radio-chemotherapy [4,5,15]. MRI findings usually consist on new contrast enhancing mass on T1-weighted imaging with gadolinium, which is indistinguishable from tumour progression. It occurs usually within the irradiated tumour volume but radiation-induced lesions could be located less commonly in the controlateral hemisphere, remotely from the primary tumour site [6]. To our knowledge, it is the first case of pseudo-progression fully described after exclusive proton RT. Here, time of occurrence was delayed (6 months) since the typical time frame is rather within 3 months after chemo-radiation, possibly due to the absence of concomitant chemotherapy [3,5,6]. Cerebral injury has been classified according to time to appearance after RT [8] as "acute", with brain oedema occurring during RT, mostly transient and reversible; subacute or "early delayed" injury, occurring a few weeks to three months post-RT, improving within six weeks; late injury, occurring months to years post-RT, including true radionecrosis. It is often irreversible, progressive, and possibly lethal and constitutes the major dose-limiting clinical complication of brain irradiation, particularly in children. Pseudo-progression (PsP) could be considered as a pathological continuum between acute post-RT reaction and treatment-related necrosis [6]. Mechanisms behind these events are not fully understood but are probably related to micro-vessels changes. Post-RT brain injury induces a focal tissue reaction with inflammatory component and increased capillary permeability, causing fluid transudation into the interstitial space and brain oedema. Transient alterations in the blood-brain barrier may be responsible for new or increased contrast enhancement, falsely suggesting tumour progression, with low perfusion values [11].

### Role of PET and advanced MR techniques in the diagnosis of "pseudo-progresion", clinical implication

This DSC-MR technique evaluates tissue changes, close to the physiopathology of PsP, whereas metabolic tools as FDG-PET or MR spectroscopy (MRS) analyse level of glucose uptake or distribution of metabolites peaks. MRS can distinguish recurrent true necrosis from active tumour [16-18] but it needs long acquisition time. In ^18^FDG-PET, FDG uptake is not specific of tumour tissue and false positive can be observed in non malignant inflammatory processes. The use of aminoacid tracers as ^11^C-methionine better discriminates post-RT necrosis from tumour recurrence [19] but it is rarely available in daily practice. Recently, the DSC-MR technique was proposed for adult high-grade gliomas in this situation [20,21] as an alternative tool. It can be incorporated in the routine follow-up, within the interval of three to six months post-RT where PsP can be observed. Importantly, there is a risk of including children with gliomas in phase II studies for "recurrent disease" that would be spontaneously reversible, leading to false positive results, consequently delivering salvage treatments for "pseudo-progressive" patients who could be, at the contrary, potentially long term survivors [15].

## Conclusion

The lack of typical clinical symptoms and the limits of anatomical MRI techniques imply that any subacute change suggesting disease progression may be considered as possible PsP. Post-RT therapy should not be interrupted too early [22] and DSC-MRI could help for distinguishing PsP from real progression.

## Consent

Written informed consent was obtained from the parents and the patient for publication of this case report and accompagnying images. A copy of the written consent is available for review by the Editor-in-Chief of this journal.

## Competing interests

The authors declare that they have no competing interests.

## Authors' contributions

CM collected the data and wrote the manuscript. FD analysed the imaging studies and contributed to the legend of figures and correction of the manuscript. DD created and developed the software used to treat T2* acquisition and produce the images. JLH supervised the irradiation of the patient. PV analysed the histology of the tumour. CSR operated the patient. CD was the treating physician of the patient. JG supervised the manuscript. All authors read and approved the final manuscript.
